# Chronic Gastritis of Long Standing, with Periodic Attacks of Migraine

**Published:** 1896-07

**Authors:** Geo. A. Curriden

**Affiliations:** Chambersburg, Pa.


### CHRONIC GASTRITIS OF LONG STANDING, WITH PERIODIC ATTACKS OF MIGRAINE. REPORT OF A CASE.*

BY GEO. A. CURRIDEN, M. D., OF CHAMBERSBURG, PA.

The herewith reported case is one of double interest, inasmuch as the patient has been under my care for a number of years, and previous to the commencement of the present treatment, I have been unsuccessful in affording much relief or preventing the recurrence of the frequent and periodic attacks of migraine, to which she had been more or less subject since early womanhood, the cause of which I could not account for more than "a habit long continued," aggravated by gastric catarrh.

The history of the case is briefly as follows: Mrs. A., age 55, since early womanhood has been subject to periodic attacks of migraine at intervals of two, three or four weeks, but seldom free from them for longer intervals.

An attack comes on by general malaise of usually a day's duration, repugnance of food or drink, marked drowsiness, much depression, with request for rest and quiet, followed by complete physicial prostration, dull frontal headache, which the least noise or disturbance makes the more intense, invariably accompanied by violent and frequent attacks of vomiting and retching, inability to retain any food or nourishment of any kind, retention of bowels, often cold sweats, pulse somewhat slow and weak and small in volume. This condition lasting usually two days, followed by gradual cessation of symptoms.

During the whole period of usually four or five days duration, she is unable to take nourishment of any kind, remains constantly in bed, and desires only complete rest and quiet. The previous treatment has been so varied and on so many different plans, that I refrain from mentioning them.

Two years ago I was able to prevent an attack for over two months by the use of strychnine in 1-20 grain doses t. i. d. with careful diet and artificial digestive.

In May, 1895, I put her on Charles Marchand's "glycozone" in teaspoonful doses well diluted t. i. d., using this, as all other previous remedies, experimentally; she commenced to

*Published by *The Medical Summary*, of Philadelphia, Pa., for March, 1896.

improve much in general health, an unusually good appetite, without the previous distressing symptoms following, a more regular movement of the bowels, freedom from headache, and in every way a decided improvement; this improvement and enjoyment of good health lasted during continuation of above treatment for over three months. Unknown to me she stopped taking the glycozone, thinking herself perfectly well. In a few weeks she had a return attack, milder and devoid of gastric distress. A similar attack two months later, both of which occurred some weeks after stopping the above described treatment, and I might say caused by imprudence in diet.

The conclusion come to in this case is that the headache is sympathetic, that the stomach becomes acutely inflamed by its inability to naturally and properly perform its functions, and responds to the call of nature to unload itself, and thus secure for a time rest, that the use of glycozone has corrected the existing gastritis and by so doing has removed the primary cause of these many years of suffering.

